# High-Speed Detection of X‑ray Pulses Using a Digital Counter Coupled with Perovskite Composite Scintillators

**DOI:** 10.1021/acsami.5c26355

**Published:** 2026-04-01

**Authors:** Samiya Khaliq, Murilo C. Faleiros, Li Zhang, Bashir E. Hasanov, Jose I. de Oliveira Filho, Mehmet Bayindir, Osman M. Bakr, Khaled N. Salama, Omar F. Mohammed

**Affiliations:** † Computer, Electrical, and Mathematical Sciences and Engineering (CEMSE) Division, 127355 King Abdullah University of Science and Technology (KAUST), Thuwal 23955-6900, Saudi Arabia; ‡ Center of Excellence for Renewable Energy and Storage Technologies, Division of Physical Science and Engineering, 127355 King Abdullah University of Science and Technology (KAUST), Thuwal 23955-6900, Saudi Arabia; § Physical Science and Engineering (PSE) Division, King Abdullah University of Science and Technology (KAUST), Thuwal 23955-6900, Saudi Arabia; ∥ Center for Hybrid Nanostructures, University of Hamburg, Hamburg 22761, Germany

**Keywords:** high-speed X-ray detection, pulse counting, perovskite composites, scintillators, high-frequency, FPGA, X-rays

## Abstract

Accurate pulse-resolved detection of ionizing radiation at megahertz frequencies is essential for applications such as quality assurance in ultrahigh-dose-rate radiotherapy and low-dose X-ray monitoring. Conventional scintillator-based detectors employ bulky single crystals, such as lutetium–yttrium oxyorthosilicate (LYSO), which limit their flexibility and integrability. Furthermore, many perovskite scintillators exhibit afterglow, which leads to signal pile-up under high-flux conditions. To address these challenges, we developed thin polymer composite scintillator films comprising LYSO and (PEA)_2_PbBr_4_. These films retained the materials’ intrinsic decay times (∼37 and ∼6 ns, respectively) while enhancing signal output through the optical scattering of scintillation photons within the inhomogeneous polymer matrix. When coupled with silicon photomultipliers and a field-programmable gate array (FPGA)-based digital counter, these films enabled rapid real-time detection across a broad frequency range. Specifically, the LYSO/PMMA composite detected intense signals up to 2 MHz (500 ns spacing), whereas the (PEA)_2_PbBr_4_/PMMA composite, with an amplification stage, enabled accurate pulse counting up to 5 MHz (200 ns spacing). With a dead time of ∼20 ns, the system resolved nanosecond-spaced pulses without pile-up, enabling reliable pulse-by-pulse readout from a few counts per second to multimegahertz bursts. These results demonstrate that inhomogeneous composite scintillator films, when integrated with FPGA-based digital processing, provide a compact and scalable pulse counter for high-frequency radiation detection, effectively addressing the limitations of conventional bulky crystal detectors.

## Graphical abstract

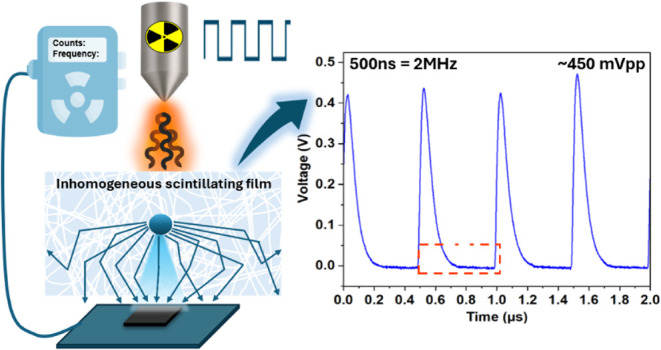

## Introduction

Radiation systems have progressed from quasi-continuous delivery and integrated readouts to time-structured beams^1^ and single-photon-sensitive electronics.^2–4^ Their performance depends not only on the radiation dose but also on the precise timing of the pulse arrival. Thus, pulse timing governs image quality,^5–9^ dosimetric precision,^10–14^ synchronization with moving targets,^15–17^ and safety margins under dynamic conditions.^18–20^ At facilities such as the European X-ray Free-Electron Laser Facility (XFEL),^21–23^ where each 10 Hz pulse train may contain up to 2,700 X-ray pulses delivered at 4.5 MHz (≈220 ns spacing), experiments routinely require pulse-by-pulse intensity monitoring synchronization.^24–30^ Further, in synchrotron and XFEL applications, adaptive gain detectors such as AGIPD and JUNGFRAU^31–36^ are specifically engineered for megahertz pulse trains, making per-pulse metrology a foundational requirement for experimental control and performance assessment.

In ultrahigh-dose-rate (UHDR/FLASH) radiotherapy, electron or photon beams are delivered in microsecond pulses at repetition rates of approximately 100–400 pulses per second (PPS) with very high dose per pulse. Consequently, pulse-resolved monitoring, rather than time-averaged dosimetry, is essential for precision and clinical quality assurance (QA), as recommended by standards bodies such as the American Association of Physicists in Medicine (AAPM TG-359).^12,37^ In photon-counting computed tomography, direct-conversion detectors operate at high instantaneous flux, where pile-up and dead time may distort counts and spectra if photons arrive within the recovery window of the electronics.^38–41^ This limitation underscores the need for fast, low-dead-time readouts capable of discriminating submicrosecond-spaced events. Mitigating such rate-related distortions is critical for ensuring accuracy under clinically relevant exposure conditions.^42,43^ In industrial security and nondestructive testing (NDT), dual-energy cargo scanners alternate the pulse energy of linear accelerators on a per-pulse basis at repetition frequencies ranging from hundreds of hertz to kilohertz to enable material discrimination, with QA and system control depending on the accurate identification and timing of individual pulses.^44–46^ Portable pulsed X-ray generators for NDT and explosive ordnance disposal emit 10–50 ns X-ray pulses at tunable repetition rates of approximately 10–25 pulses per second and similarly require precise per-pulse measurement to ensure output verification and operational safety.^47,48^

High-frequency radiation pulse counters provide the temporal resolution required to distinguish closely spaced events and verify the stability of individual pulses across clinical, industrial, and large-scale scientific applications. Although standard pulse counters are effective, they encounter limitations such as pile-up and dead time, which cause loss of counts and upward bias in energies when events occur within the recovery window of the detector or electronics.^42,43^ QA systems depend on pulse-resolved dosimetry to capture dose-per-pulse stability and identify anomalies, as integrating monitors may fail to detect pulse-to-pulse fluctuations in UHDR beams.^37^ Standard leading-edge discriminators introduce timing errors, known as time-walk, wherein the detected time shifts with the pulse amplitude. These errors distort timing accuracy and introduce jitter unless corrected using constant-fraction discriminators or algorithmic methods, such as those employed in positron emission tomography (PET) detectors.^49,50^

Although Geiger–Müller (GM) tubes are valued for their simplicity, they exhibit fundamental limitations in applications requiring high-count-rate linearity, energy discrimination, or precise timing. Their primary constraint is extended dead time, which can range from tens to hundreds of microseconds and is highly dependent on the operating voltage. Under varying voltages, it may reach a minimum of approximately 9 μs at low bias but increase to approximately 292 μs at higher voltages, leading to count-rate saturation and nonlinear response at high flux.^51,52^ Meanwhile, other detectors, such as high-purity germanium detectors, exhibit a dead time of approximately 5–10 μs.^53^ Consequently, GM tubes require complex correction methods to achieve accuracy, rendering them unsuitable for applications demanding high linearity, energy resolution, and a precise temporal response.

Hybrid scintillators are well-known for their efficiency in X-ray detection and imaging.^54,55^ However, scintillation-based counters face intrinsic challenges linked to afterglow.^56^ When exposed to high-rate or bursty radiation, the slow decay components of scintillator emission elevate the baseline between pulses, resulting in pile-up and reduced temporal resolution.^56^ In high-rate environments where pulses are separated by microseconds or less, this overlap hinders accurate event discrimination, and the detector cannot distinguish overlapping events accurately.^57^ Residual luminescence further increases the effective dead time, constraining the detection range and preventing a precise linear response.^58^ These effects can trigger false signals in pulse-processing electronics, increase thresholds, and diminish sensitivity to weak signals.

To overcome these limitations, we developed an improved, high-frequency pulse detection system integrating a field-programmable gate array (FPGA) with fast-decaying and low-afterglow scintillators: lutetium–yttrium oxyorthosilicate (LYSO) and (PEA)_2_PbBr_4_.^59,60^ As depicted in Figure 1a, pulsed X-ray excitation generates photons in inhomogeneous scintillation films, which are collected by a SiPM and converted into electrical signals. These signals are processed in real time by the FPGA, enabling accurate discrimination and preventing pile-up, even for nanosecond-spaced events. The processed counts per second and radiation frequency data are transmitted to a microcontroller, which displays the results on an organic light-emitting diode (OLED) module, while raw waveforms are simultaneously displayed on an oscilloscope for verification. Figure 1b and c illustrates the comparison of photon transport between a homogeneous scintillator crystal and an inhomogeneous scintillating film. In the homogeneous crystal (Figure 1b), a substantial fraction of the emitted photons undergo total internal reflection at the film surface, leading to extensive light trapping and reduced photon transmission to the detector. In contrast, the inhomogeneous scintillating film (Figure 1c) introduces internal scattering events that redirect a considerably greater number of photons toward the SiPM. This enhanced photon delivery to the SiPM, coupled with spectral matching^61^ and alongside the intrinsically fast scintillation response,^62^ allows stable baseline recovery and pulse-by-pulse resolution when excitation pulses arrive only a few nanoseconds apart (∼20 ns), ensuring reliable QA and safety across a range of pulse-by-pulse resolution conditions, ranging from microseconds to multi-MHz bursts. The integration of fast scintillators, SiPM, FPGA-based signal processing, and microcontroller display addresses a key limitation in high-frequency radiation pulse detection, enabling reliable operation for high-flux applications.

**Figure 1:**
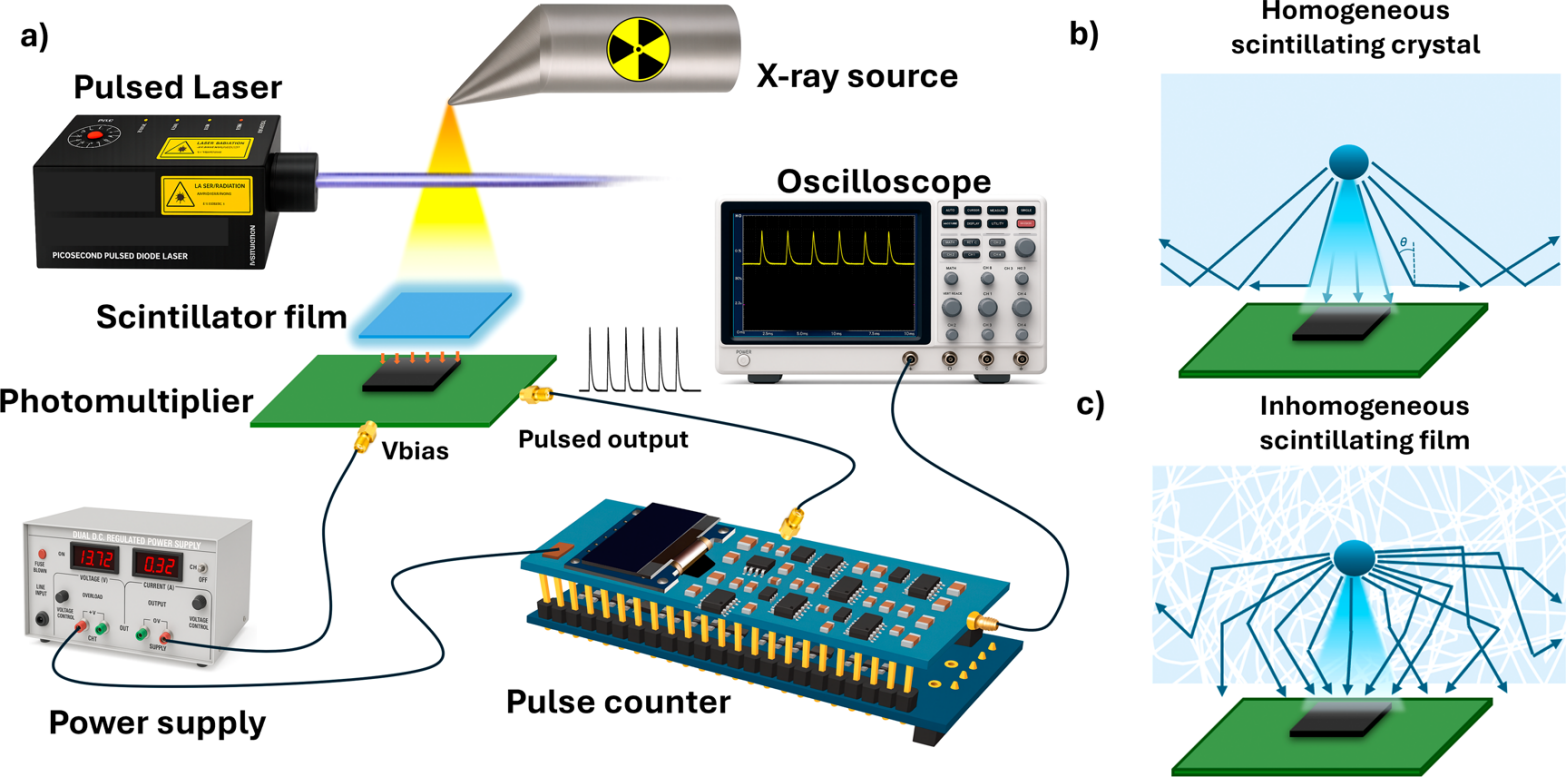
Schematic of a scintillation-based pulse detection and counting system. (a) System setup including a pulsed laser source, an X-ray source, a scintillator film, and a silicon photomultiplier (SiPM) powered by a DC bias supply (*V*_bias_). The amplified pulsed output is processed and displayed using a pulse counter board and an oscilloscope. (b) Homogeneous scintillating crystal under X-ray excitation. (c) Inhomogeneous composite scintillating film under X-ray excitation.

## Experimental Section

### Inhomogeneous LYSO and (PEA)_2_PbBr_4_ Scintillator Films

Selecting scintillating materials with fast decay times and high light yields is essential for detector design, as the signal-to-noise ratio (SNR) depends on the number of detected photons *N*, following the relation $SNR=\sqrt{N}$ . In this context, increased light yield raises *N*, thereby improving the SNR and detector performance. Furthermore, fast decay arises from efficient processes, leading to minimal to no afterglow and pile-up, while high light yields ensure efficient photon conversion and high-amplitude signals for SiPM readout; together, these parameters determine the system’s temporal resolution and counting reliability.^58,63^ Conventional detectors often employ bulk LYSO crystals, which provide excellent light yield and nanosecond-scale decay but are bulky and rigid, limiting the integration flexibility. In previous studies, such crystals have been fabricated with large dimensions (e.g., ⌀64 mm × 220 mm, weighing several kilograms) for calorimetry and PET-grade applications.^64,65^ Similarly, long-form LYSO crystals (∼3 × 3 × 20 mm^3^ and larger arrays) have been standard in time-of-flight positron emission tomography detectors, where they are assembled in rigid, segmented formats (e.g., 8 × 8 arrays of 3.1 × 3.1 × 20 mm^3^; 16 × 16 arrays of 1.5 × 1.5 × 20 mm^3^).^66^ Another limitation of bulk inorganic scintillators is their intrinsically high refractive index, attributed to the material density required to effectively attenuate ionizing radiation. Further losses arise from optical coupling at the interface, where Fresnel reflections reduce the photon transmission. No optical coupling medium was applied in these measurements to maintain a direct and consistent comparison between the composite films under identical conditions. More critically, the total internal reflection (TIR) within the single crystal traps scintillating light, allowing only a small fraction ( $\eta =(1-\sqrt{(1-1/n^{2})/2}$ , where *n* is the refractive index of the scintillator) of emitted photons to reach the detector.^67^ Specifically, due to the high refractive index of LYSO (*n* = 1.82), only 8.2% of the scintillation light reaches the detector.

To address these limitations, we fabricated inhomogeneous LYSO/PMMA and (PEA)_2_PbBr_4_/PMMA composites by grinding bulk crystals and recasting them into films (Supporting Information). The composite films had a thickness of 500 μm, matching that of the corresponding bulk crystal samples to enable a direct performance comparison. Notably, both composites emit intense radioluminescence under X-ray excitation (Figure 2a and 2d), with corresponding spectra (Figure 2b and 2e) indicating apparent light yield enhancements of 2.08 times for LYSO (corresponding light yield of 91,388 photons/MeV) and 1.6 times for (PEA)_2_PbBr_4_ (corresponding light yield of 34,553 photons/MeV) relative to their single-crystal counterparts of equivalent thickness. This significant enhancement is driven by a profound increase in the light extraction efficiency. By embedding the high-index scintillating grains into the lower-index PMMA matrix (*n* = 1.50),^68^ we introduce localized refractive index gradients that induce strong optical scattering. This scattering efficiently breaks the total internal reflection, providing trapped photons with new geometric escape pathways. Consequently, the composite architecture successfully rescues scintillation light that would otherwise be permanently trapped within the dense LYSO (*n* = 1.82) and (PEA)_2_PbBr_4_ (*n* = 2.13)^69^ crystal structures. Interestingly, the ratio of these net enhancement factors (2.08/1.60 = 1.30) scales almost perfectly with the ratio of their measured X-ray attenuation efficiencies (0.73/0.56 ≈ 1.30) at 50 keV. This direct correlation demonstrates that the inhomogeneous PMMA matrix provides a highly consistent optical extraction boost across both materials. The difference in the final photon yield enhancement is therefore fundamentally governed not by variations in optical scattering efficiency but by the higher heavy-metal mass fraction in the LYSO film available to absorb the primary X-ray dose.

**Figure 2:**
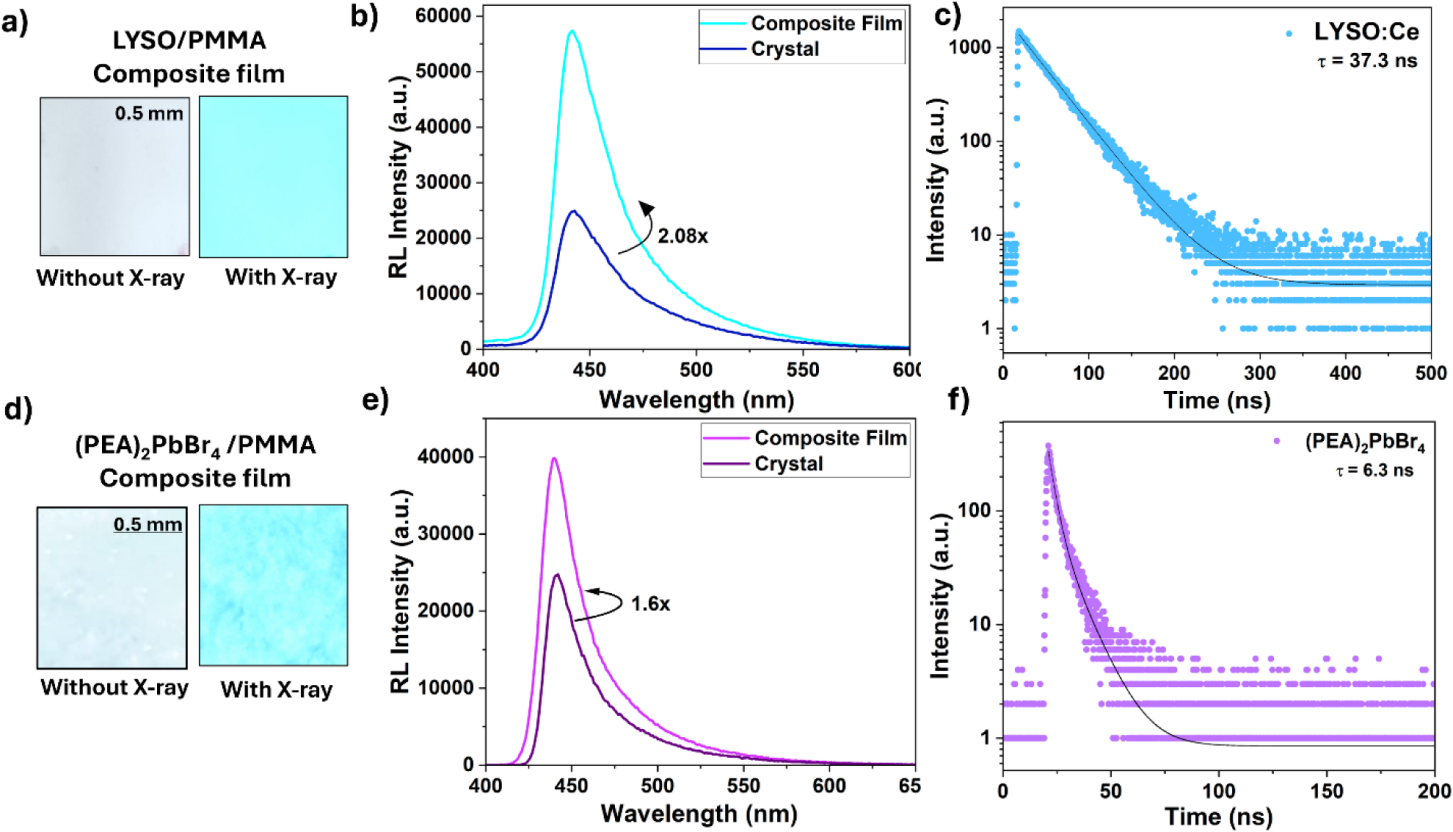
Inhomogeneous LYSO and (PEA)_2_PbBr_4_ scintillator films. (a) Photographs of the LYSO/poly­(methyl methacrylate) (PMMA) composite film under X-ray excitation, displaying blue emission with a peak near 445 nm. (b) Radioluminescence spectra of the LYSO/PMMA film and crystal, showing that the film produces approximately 2.08 times higher light output compared to the crystal. (c) Time-resolved decay of LYSO:Ce crystal under X-ray irradiation, exhibiting a single-component decay with a lifetime τ = 37.3 ns. (d) Photographs of the (PEA)_2_PbBr_4_/PMMA composite film under X-ray excitation showing a blue emission peak near 445 nm. (e) Radioluminescence spectra of the (PEA)_2_PbBr_4_/PMMA film and crystal, indicating that the film yields 1.6 times higher light output as compared with the crystal. (f) Time-resolved decay of the (PEA)_2_PbBr_4_ crystal under X-ray irradiation, exhibiting an exponential decay with τ = 6.3 ns.

We also synthesized composites with varied particle sizes and material-to-polymer ratios (Figure S1a and S1b). Notably, larger particles generate stronger scattering and retain a higher absorption volume, yielding the highest extraction efficiency, as observed for the 0.46 mm LYSO composite. As the particle size decreases, the reduced scattering strength and lower absorption volume lead to diminished light extraction. This illustrates the trade-off between enhancing photon extraction via scattering and preserving sufficiently large grains for effective X-ray energy deposition.^70^ A similar compositional optimization was conducted for the (PEA)_2_PbBr_4_/PMMA composites (Figure S2). The 300:600 mg (PEA)_2_PbBr_4_:PMMA film with large particles exhibited higher radioluminescence than the 600:600 mg compositions. This behavior reflects a balance between increased X-ray attenuation at higher material loadings and enhanced photon self-absorption and optical transport losses within the perovskite domains. Therefore, the 300:600 mg large-particle composite represents a better balance between radiation attenuation and optical extraction efficiency, resulting in a higher RL output.

Notably, the decay times remain unchanged relative to the bulk crystals, which are 37 ns for LYSO (Figure 2c) and 6 ns for (PEA)_2_PbBr_4_ (Figure 2f), with all composite films exhibiting lifetimes ranging from 30 to 37 ns (Figure S3a and S3b), thereby maintaining fast scintillation characteristics.^71^ When the X-ray response speed of LYSO:Ce and undoped PEA_2_PbBr_4_ is evaluated, both the rise time and emission decay must be considered. For LYSO:Ce, Gundacker et al.^72^ demonstrated using pulsed X-ray excitation that the rise time is best described by a two-component model: a dominant fast component effectively at the system resolution limit with <10 ps and a slower component of approximately 300 ps carrying an effective single-component approximation of ∼70 ps depending on the crystal. For undoped PEA_2_PbBr_4_, Cala et al.^73^ measured the scintillation kinetics under the same X-ray energy regime (0–40 keV, mean ∼9.15 keV) and found the rise time to be below the system detection threshold, reporting it as <30 ps due to the ultrafast self-trapped exciton (STE) formation driven by the strong electron–phonon coupling within the [PbBr_6_]^4-^ inorganic layers. PEA_2_PbBr_4_ shows no evidence of a slow rise component, unlike LYSO:Ce, while also offering a faster effective decay time; therefore, these results suggest that undoped PEA_2_PbBr_4_ offers a faster and cleaner temporal response under X-ray excitation than standard LYSO:Ce, though this advantage must be studied against its considerably lower X-ray stopping power and less light yield. Therefore, it is worth mentioning that the rise time is much faster than both the decay time of the scintillators and the SiPM recharge time, making it not a constraint for the pulse counting experiments.

The SEM images show distinct particle sizes for (PEA)_2_PbBr_4_ and LYSO. Specifically, the average size is 26.8 μm for (PEA)_2_PbBr_4_, 12.68 μm for large LYSO:Ce particles, and approximately 2.52 μm for small LYSO:Ce particles, as indicated in Figure S4a–c. These microstructural variations influence scattering behavior and, consequently, the light yield enhancement observed in the composite films. Figure S5a presents the measured attenuation coefficients of (PEA)_2_PbBr_4_ and LYSO:Ce, indicating that the perovskite exhibits higher attenuation at low-to-intermediate photon energies (<100 keV) and thus demonstrates more efficient X-ray absorption. Further, as depicted in Figure S5b, nearly complete attenuation (100%) is achieved at 50 kV and 0.5 mm thickness for both LYSO:Ce and (PEA)_2_PbBr_4_/PMMA films. Because our experimental setup is currently limited to photon energies below 50 keV, we considered the expected scintillation behavior in the 20–50 kV range, which is relevant for applications such as dental radiography. In this context, nonproportionality of the light yield becomes an important factor for an accurate energy response. LYSO:Ce is known to exhibit substantial nonproportionality, with an overall variation of ∼35% across 16.6–1274 keV (normalized to 662 keV), with the most pronounced deviation occurring at low energies around 16.6 keV, indicating strong energy dependence of the scintillation yield.^74^ In contrast, undoped (PEA)_2_PbBr_4_ shows comparatively small light-yield variation between 59.5 and 662 keV, although its energy resolution remains strongly excitation-energy dependent, confirming the presence of nonproportional effects.^75,76^ These observations suggest that within the 50–200 keV range, (PEA)_2_PbBr_4_ is expected to exhibit reduced light-yield nonlinearity compared to LYSO:Ce, though careful calibration remains necessary for precise energy discrimination in practical dosimetric applications. Thus, transforming bulky crystals into thin composite scintillator films enables a form factor suitable for integration with high-frequency counters while simultaneously enhancing the light yield and preserving ultrafast decay characteristics. Notably, even at the minimum X-ray tube setting (40 kV, 0.1 μA), the LYSO/PMMA film generated a measurable peak signal of approximately 150 mV (Figure S6), confirming adequate sensitivity under low-flux conditions. Together, these advantages position the LYSO/PMMA and (PEA)_2_PbBr_4_/PMMA as highly effective composites, promising candidates for megahertz-rate pulse-by-pulse radiation monitoring.

To evaluate the suitability for practical applications, the stability of the (PEA)_2_PbBr_4_/PMMA composite films was assessed as a function of delivered dose and humidity. After irradiation with more than 600 mGy, the composite retained approximately 98.9% of its initial radioluminescence intensity, as shown in Figure S7a, indicating that doses substantially higher than those encountered in routine medical diagnostic imaging do not produce significant defect-related luminescence degradation. For context, typical absorbed doses from common diagnostic radiography examinations are on the order of less than a few mGy per exposure,^77^ and even computed tomography (CT) examinations generally involve absorbed doses in the single- to low-tens of mGy range to the organs of interest, far below 600 mGy of localized exposure in our tests.^78^ Furthermore, humidity tolerance was also examined by submerging the film in water and irradiating repeatedly at 40 kV and 80 μA. After 1 h under these harsh conditions, the film maintained approximately 86% of its initial radioluminescence, as shown in Figure S7b, demonstrating that the PMMA composite film can effectively resist extreme humidity without significant loss of scintillation performance.

## Results and Discussion

### X-ray Pulse Detection System

The detection system comprises a pulsed X-ray source, a LYSO/PMMA–SiPM detector assembly, and an FPGA-based digital pulse counter. As shown in Figure 3a, the composite LYSO/PMMA scintillator is coupled to a 6 mm SiPM (microcell size of 35 μm and a recharge time constant ∼95 ns). The SiPM recharge time constant refers to the intrinsic recovery time of a microcell after avalanche breakdown, whereas the system dead time denotes the minimum time interval required to resolve two consecutive pulses in the readout electronics. For comparison, the direct response of the bare SiPM under identical X-ray conditions (40 kV, 1.5 μA, 500 ns pulse spacing) is provided in Figure S8, demonstrating that scintillator coupling enhances the signal amplitude by more than 20-fold. Additionally, a direct comparison between a 500 μm LYSO:Ce crystal and a 500 μm LYSO:Ce/PMMA composite film under identical low-dose pulsed X-ray conditions is shown in Figure S9. Notably, a low X-ray dose of 45.05 μGy/s (40 kV, 0.3 μA) was intentionally used to minimize saturation effects and ensure that the measured signal differences reflect intrinsic photon extraction efficiency rather than detector nonlinearity.

**Figure 3:**
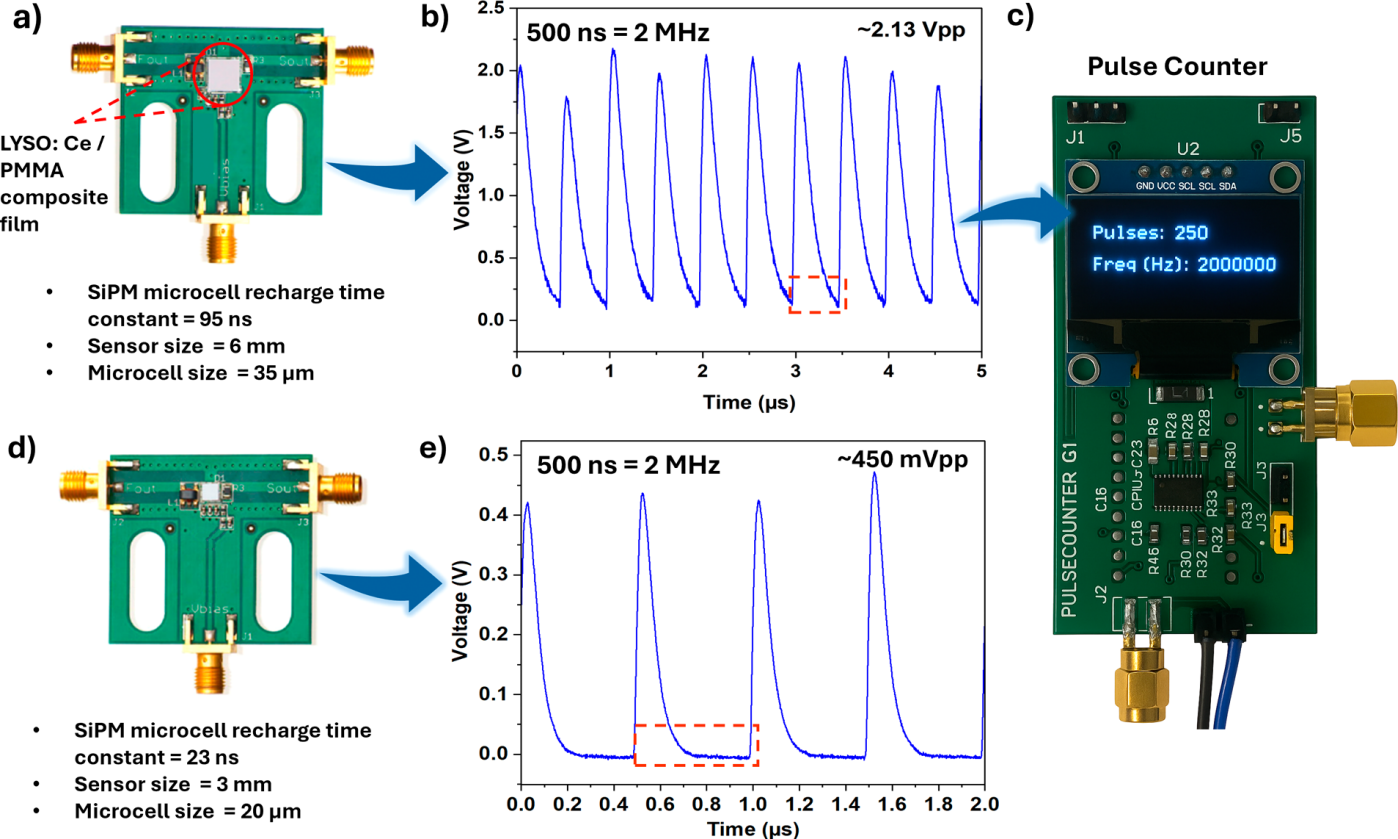
Performance comparison of two SiPM-based detection setups integrated with a high-frequency pulse counter: (a) Setup incorporating a 6 mm SiPM with 35 μm microcells and an LYSO:Ce/PMMA composite scintillating film. The SiPM exhibits a microcell recharge time constant of 95 ns. (b) Output waveform from setup (a) under a 2 MHz pulsed input (500 ns interval), exhibiting a ∼2.13 V peak-to-peak voltage (*V*_pp_). (c) Display output from the pulse counter interfaced with setup (a), indicating the detection of 250 pulses and a frequency of 2 MHz. (d) Setup incorporating a 3 mm SiPM with 20 μm microcells and a recharge time constant of 23 ns. (e) Output waveform from setup (d) under a 2 MHz pulsed input, showing a ∼450 mV peak-to-peak voltage, attributed to the shorter recharge time and smaller microcell size of the sensor.

Under identical conditions, the composite film exhibits a peak voltage approximately 1.6 times that of the bulk crystal. Since both samples have the same thickness (500 μm) and were placed in direct contact with the same SiPM, the increased signal amplitude directly reflects improved photon extraction and collection efficiency in the inhomogeneous structure. The enhancement arises from internal scattering within the polymer matrix, which reduces the total internal reflection and redirects scintillation photons toward the detector. Since both samples were measured under identical geometry and SiPM coupling, the enhancement confirms improved photon extraction efficiency in the inhomogeneous composite.

The SiPM lacks an internal amplifier; it is connected to a two-stage noninverting AD8000YRDZ amplifier, which amplifies its millivolt-level output to a *V*_pp_ of approximately 2.13 V, thereby ensuring compatibility with the CMOD A7 FPGA. To enable high-frequency counting, a 2-bit asynchronous frequency divider is implemented to divide the frequency of the raw pulse train by four. The most significant bit (MSB) of the asynchronous counter is subsequently fed into a synchronized rising-edge counter clocked at 100 MHz. This configuration offers a theoretical detection limit as low as 2.5 ns for the raw pulse widths. However, constraints in the input/output pin speed raise the actual detection limit beyond this value. The synchronized rising-edge counter is reset every 500 μs, resulting in a minimum detectable pulse train frequency of 2 kHz, which can be adjusted by modifying the counter bit width or reset interval. During each 500 μs reset period, the total number of pulses is recorded as a sample of the pulse train frequency. The recorded data are transmitted via a UART-TX module as MSB and least significant bit values; these values are then reconstructed by ATmega into pulse counts and frequency data before updating the OLED display over SPI.

The system dead time is governed not by the counter itself but by the interface protocol. Employing a communication protocol with a bandwidth above 2 kHz × 16 bits (i.e., 32 kb/s) reduces transmission overhead, enabling the dead time to approach the counter synchronization level, equivalent to just two clock cycles (∼20 ns in this implementation). Here, under 2 MHz pulsed excitation, the SiPM produced periodic waveforms (Figure 3b), which were concurrently captured by the digital counter and displayed as 250 pulses at 2 MHz on the OLED module (Figure 3c). Because the SiPM recharge time (∼95 ns) is completed before subsequent pulses at high repetition rates. Notably, a similar limitation affects the (PEA)_2_PbBr_4_/PMMA composite film (Figure S10a), where it contributes additional dead time arising from electronic delays.

To mitigate this issue, a smaller 3 mm SiPM with a 20 μm microcell size and a reduced recharge constant (∼23 ns) was adopted (Figure 3d). As expected, the resulting output pulses (Figure 3e) preserved the 2 MHz periodicity but exhibited a lower amplitude (∼450 mV_pp_), owing to the reduced active area and microcell capacitance. To restore signal levels, the (PEA)_2_PbBr_4_/PMMA composite film was integrated with an additional amplification stage (Figure 4a). Notably, the amplifier stage with a gain of 2× increased the signal amplitude, facilitating stable pulse detection. Under 2 MHz pulsed excitation with 500 ns spacing, the detector generated periodic pulses with a *V*_pp_ of approximately 2 V, as shown in Figure 4b. The recharge time of this SiPM was approximately 23 ns, which is shorter than the decay time of the LYSO/PMMA scintillator; consequently, the scintillation signal did not fully decay before the arrival of the subsequent pulse, increasing the likelihood of pulse pile-up, as shown in Figure S10b. When the repetition rate was increased to 5 MHz (200 ns spacing), the output voltage dropped to approximately 1 *V*_pp_ and exhibited increased jitter, as shown in Figure S10c. This diminished signal was subsequently amplified with a gain of 3× to restore an amplitude of approximately 2 *V*_pp_ for FPGA-based processing, as shown in Figure 4c. These results emphasize that selecting the SiPM based on its microcell recharge time is as critical as choosing the scintillator based on its decay time to achieve MHz or higher-range detection without pulse pile-up or baseline distortion. The processed output was displayed on the FPGA-based digital counter (Figure 4d), which accurately recorded 625 pulses at 5 MHz, confirming a real-time counting precision. To quantitatively position the performance of the present system relative to current radiation detection technologies, a structured comparison of key performance parameters, including upper detection frequency, dead time, detector form factor (volume), light output, and cost, is summarized in Table S1 (Supporting Information). Overall, these findings demonstrate that integrating an inhomogeneous composite scintillator film with FPGA-based digital processing gives a compact and scalable pulse counter suitable for high-frequency radiation detection with nanosecond resolution.

**Figure 4:**
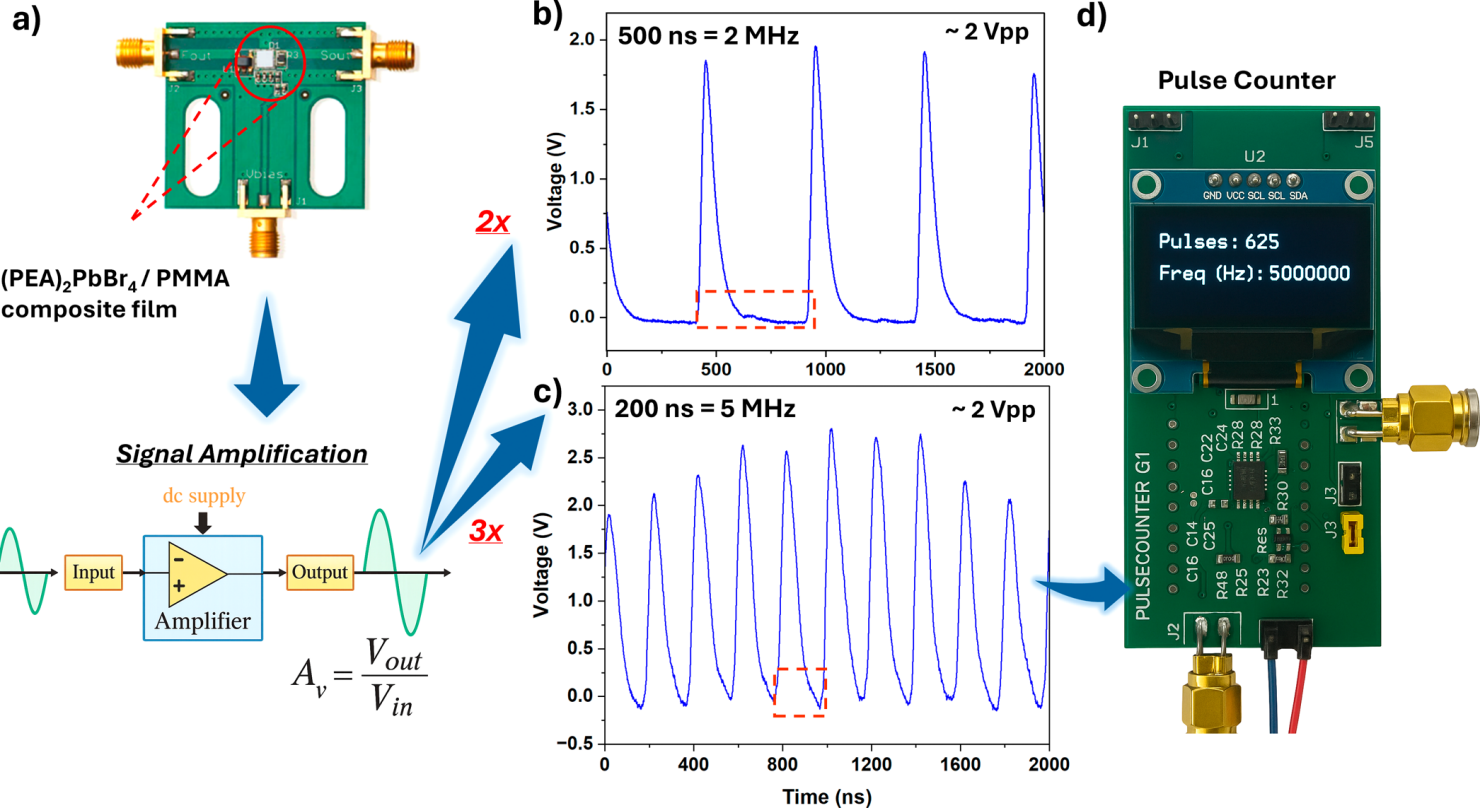
Pulse signal amplification for the (PEA)_2_PbBr_4_/PMMA composite scintillating film. (a) Output signal from the SiPM amplified in two sequential gain stages (2× and 3×) to increase pulse amplitude. (b) Output waveform under a 2 MHz input (500 ns spacing) following two-stage amplification, yielding a *V*_pp_ of approximately 2 V. (c) Output waveform under a 5 MHz input (200 ns spacing) after three-stage amplification, also reaching a *V*_pp_ of ∼2 V. (d) Pulse counter output showing the detection of 625 pulses and accurate identification of a 5 MHz input frequency.

## Conclusions

In this study, we addressed the limitations of conventional scintillator detectors in high-frequency radiation monitoring by developing and validating composite scintillator films integrated with an FPGA-based digital pulse counter. Notably, the fabricated inhomogeneous LYSO/PMMA and (PEA)_2_PbBr_4_/PMMA films retained the fast decay times of their respective parent materials (∼37 ns for LYSO and ∼6 ns for (PEA)_2_PbBr_4_) while exhibiting enhanced light yield through internal scattering within the polymer matrix. When coupled with SiPMs and FPGA-based digital counters, these films enabled reliable pulse detection at higher frequencies. The LYSO/PMMA film produced stable, high-amplitude signals at 2 MHz, whereas the (PEA)_2_PbBr_4_/PMMA film, following signal amplification, accurately detected pulses at 5 MHz (200 ns spacing). Notably, the FPGA counter distinguished individual pulses in real time with a dead time of approximately 20 ns, thereby eliminating pile-up and enabling pulse-by-pulse resolution across a wide frequency window, ranging from a few counts per second to multimegahertz bursts. These results demonstrate that thin, inhomogeneous composite scintillator films, when integrated with an FPGA-based pulse counter, offer high-frequency radiation detection. The integrated pulse counter provides the temporal precision necessary to distinguish closely spaced events and verify pulse stability in clinical, industrial, and large-scale scientific applications.

## Supplementary Material



## Notes

Synthesis of (PEA)_2_PbBr_4_ and light yield calculation; radioluminescence comparison of (PEA)_2_PbBr_4_/PMMA composite films; radioluminescence comparison of different LYSO/PMMA composite films; comparison of decay times of the composite films; SEM images with average particle sizes; absorption coefficient and attenuation efficiency of the scintillators; detection limit of the device; humidity and radiation stability studies; response of the SiPM in the absence of the scintillators; performance comparison of bulk LYSO:Ce and LYSO:Ce composite films in the SiPM; comparison of recharge times of silicon photomultipliers; comparison table of current pulse-counter technologies (PDF)
