# Supplementary material for: High-Speed Detection of X‑ray Pulses Using a Digital Counter Coupled with Perovskite Composite Scintillators
Source: ACS Appl Mater Interfaces. 2026 Apr 1;18(14):20668–77. doi: 10.1021/acsami.5c26355 (PMC13088026; doi:10.1021/acsami.5c26355)
Supplement: Supplementary file 1 [file am5c26355_si_001.pdf]

Supporting Information

High-Speed Detection of X-Ray Pulses Using a Digital Counter Coupled with Perovskite Composite Scintillators

Samiya Khaliq[a,b§], Murilo C. Faleiros[a,b§], Li Zhang[a,b], Bashir E. Hasanov[b,c], Jose I. de Oliveira Filho[a,b], Mehmet Bayindir[b,d*], Osman M. Bakr[b,c], Khaled N. Salama[a,b], and Omar F. Mohammed[b,c*]

[a]Computer, Electrical, and Mathematical Sciences and Engineering (CEMSE) Division, King Abdullah University of Science and Technology (KAUST), Thuwal 23955-6900, Saudi Arabia.

[b]Center of Excellence for Renewable Energy and Storage Technologies, Division of Physical Science and Engineering, King Abdullah University of Science and Technology (KAUST), Thuwal 23955-6900, Saudi Arabia.

[c]Physical Science and Engineering (PSE) Division, King Abdullah University of Science and Technology (KAUST), Thuwal 23955-6900, Saudi Arabia.

[d]Center for Hybrid Nanostructures, University of Hamburg, 22761 Hamburg, Germany

[§]S.K. and M.C.F. contributed equally to this work

*Corresponding Author: Omar.Abdelsaboor@kaust.edu.sa; mehmet.bayindir@kaust.edu.sa

Preparation of Non-homogeneous LYSO and $(PEA)_2PbBr_4$ Scintillating Films

Lutetium–yttrium oxyorthosilicate doped with cerium (LYSO:Ce) was purchased from OST Photonics. $PbBr_2$ (99%) was purchased from Macklin, while phenethylammonium bromide (PEABr) was purchased from Greatcell Solar Materials. For the synthesis of $(PEA)_2PbBr_4$, PEABr and $PbBr_2$ were mixed in DMF solvent in a 2:1 ratio and left to slowly evaporate over several days under ambient conditions inside a fume hood, until crystals formed at the bottom. These crystals were then collected and dried. For the synthesis of non-homogeneous films, poly(methyl methacrylate) (PMMA) was mixed in different ratios with ground crystals of LYSO or $(PEA)_2PbBr_4$, subjected to varying grinding times in a mortar and pestle, and subsequently dissolved in dichloromethane. The solution was drop-cast into a silicon mold and left to dry, forming smooth films.

For light-yield calculations, samples were held in support, and a small area of the film or crystal was left exposed to standardize the area of collection. Both films and crystals had the thickness of 500 μm. Light yield was calculated using the equation:

$$\mathrm{LY_S} = \mathrm{LY_R} \times \frac{\int \mathrm{RL_S}(\lambda)\,\mathrm{d}\lambda\,/XAE_\mathrm{S}}{\int \mathrm{RL_R}(\lambda)\,\mathrm{d}\lambda\,/XAE_\mathrm{R}}$$

where the $\mathrm{LY_R}$ is the light yield of the reference LYSO:Ce scintillator (32,000 photons/MeV, OST Photonics), while XAE_R and XAE_S are the measured X-ray attenuation efficiencies of the reference and sample, respectively. The data for these attenuation efficiencies were collected by measuring the dose rate in front of the X-ray source with (D_S) and without (D_0) a sample placed in the X-ray beam path:

$$XAE = \frac{D_0 - D_\mathrm{S}}{D_0}$$

The estimated X-ray attenuation efficiencies given below:

$XAE_{\mathrm{LYSO\ Crystal}} = 0.9972$; $XAE_{\mathrm{LYSO\ Film}} = 0.7287$

$XAE_{\mathrm{(PEA)_2PbBr_4\ Crystal}} = 0.9968$; $XAE_{\mathrm{(PEA)_2PbBr_4\ Film}} = 0.5637$

The corresponding light yields:

$\mathrm{LY_{LYSO\ Film}} =\ 91{,}388$ photons/MeV

$\mathrm{LY_{PEA)_2PbBr_4\ Film}} =\ 34{,}553$ photons/MeV

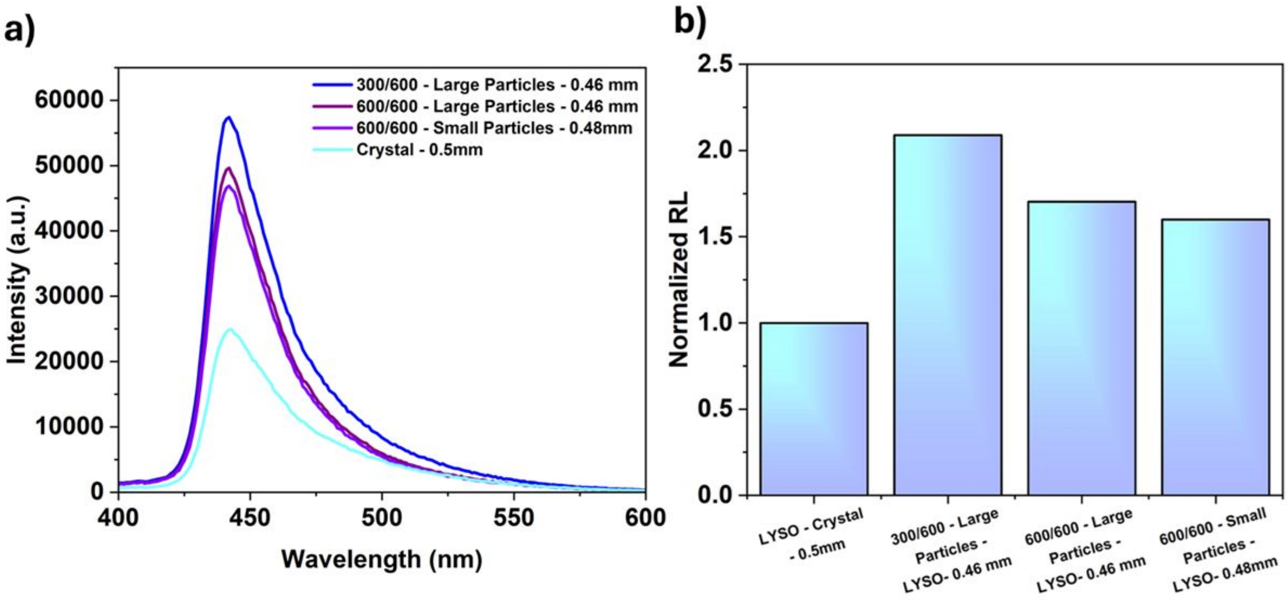

Figure S1: Radioluminescence (RL) spectra of LYSO/PMMA composite films. (a) RL spectra of LYSO/PMMA composite films with different particle sizes and material-to-polymer ratio, compared to the bulk LYSO crystal. Films exhibit much higher emission intensity than crystal, with large-particle composites showing the strongest response. **(b)** Normalized RL shows enhancement in films relative to the crystal, with particle size and material-to-polymer ratio influencing the light yield.

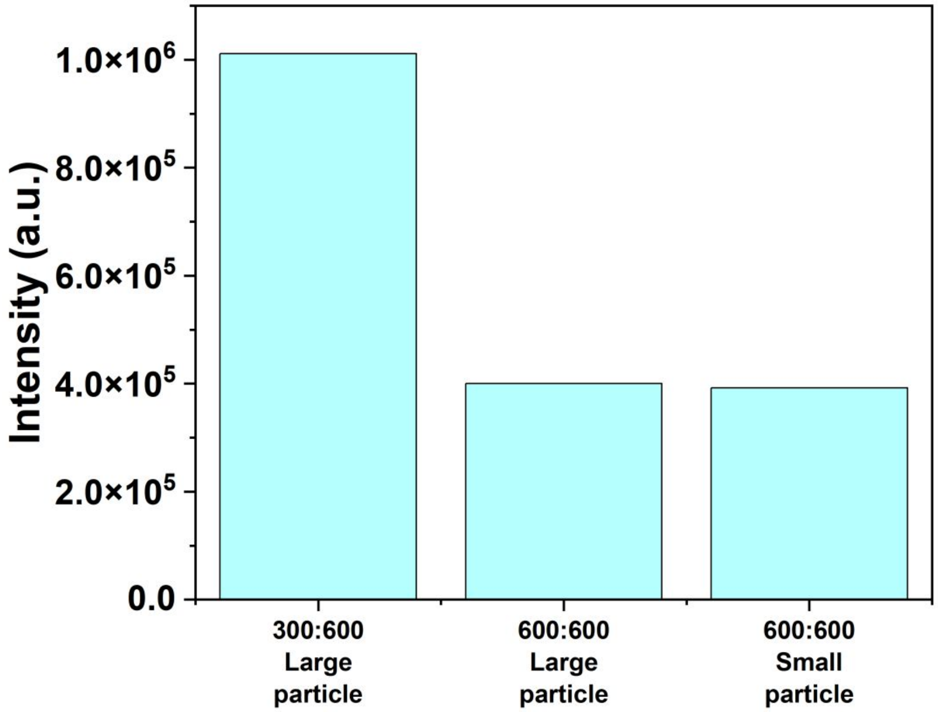

Figure S2: Radioluminescence comparison of $(PEA)_2PbBr_4$/PMMA composites. Composites with varying loading ratios and particle sizes. The 300:600 mg large-particle composite exhibits the highest intensity, indicating an optimal balance between X-ray attenuation and optical extraction efficiency compared to higher-loading configurations.

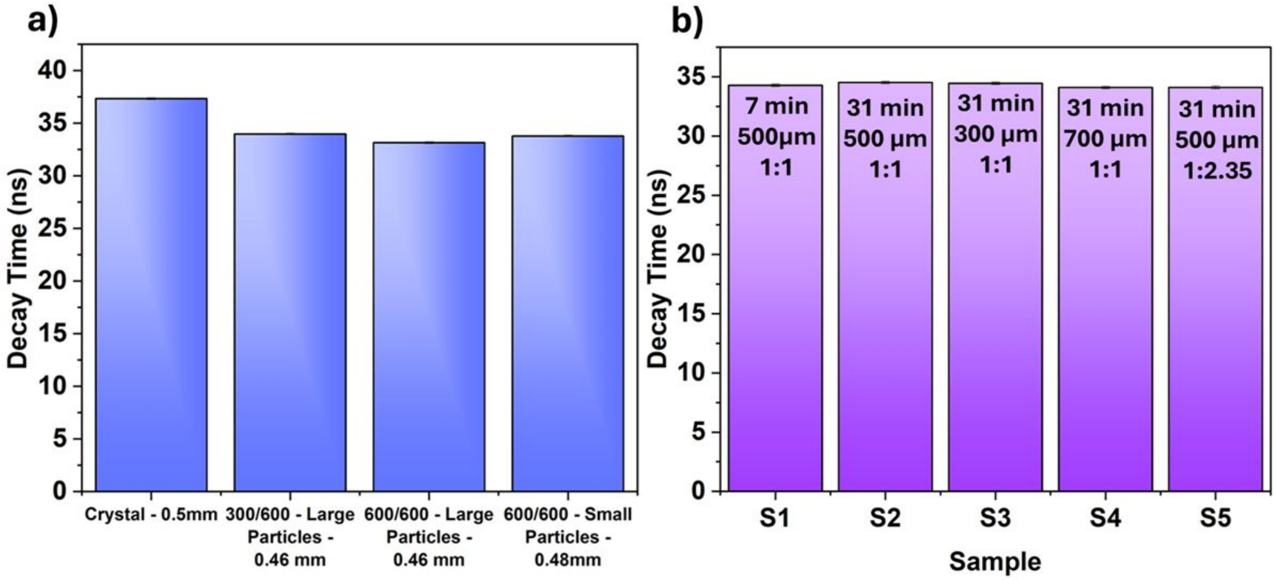

Figure S3: Decay times of LYSO/PMMA composite films. (a) Decay times of LYSO/PMMA composite films with different particle sizes and l material-to-polymer ratio compared to the crystal. All composites show lifetimes close to the crystal (~30–37 ns), preserving fast scintillation requirements. **(b)** Decay times of LYSO/PMMA films under varying fabrication conditions (grind time, particle size, and mixing ratio). All samples exhibit ultrafast lifetimes (~30 ns), with minimal variation across processing parameters.

Sample 1 - 7 min grinding, 200mg of LYSO, 200mg of PMMA, 500 microns thick

Sample 2 - 31 min grinding, 200 mg of LYSO, 200mg of PMMA, 500 microns thick

Sample 3 - 31 min grinding, 200 mg of LYSO, 200mg of PMMA, 300 microns thick

Sample 4 - 31 min grinding, 200 mg of LYSO, 200mg of PMMA, 700 microns thick

Sample 5 - 31 min grinding, 85 mg of LYSO, 200mg of PMMA, 500 microns thick

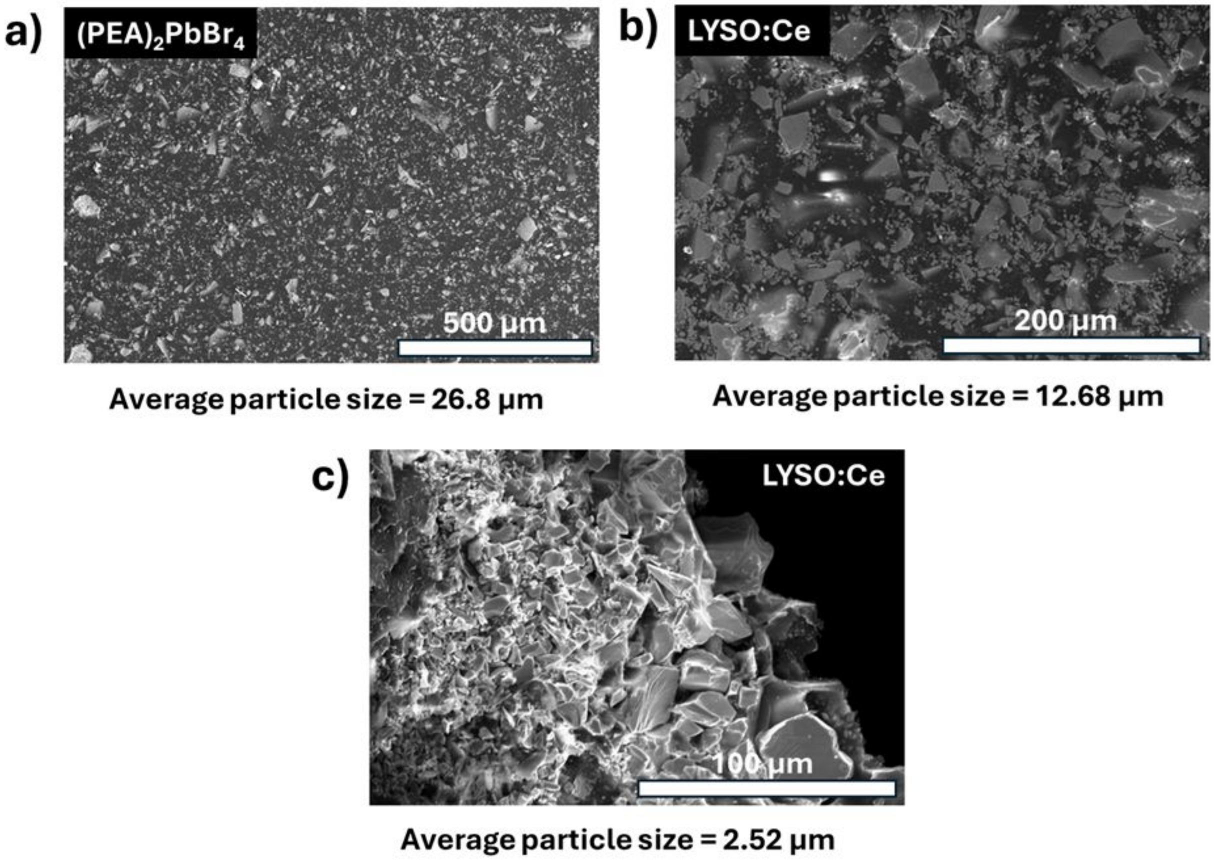

Figure S4: SEM Images of $(PEA)_2PbBr_4$ and LYSO:Ce composite films. a) $(PEA)_2PbBr_4$ particles showing a relatively coarse morphology with an average particle size of ~26.8 µm.

b) Large LYSO:Ce particles with a finer distribution, exhibiting an average size of ~12.68 µm.

c) Small LYSO:Ce particles exhibit an average particle size of ~2.52 µm.

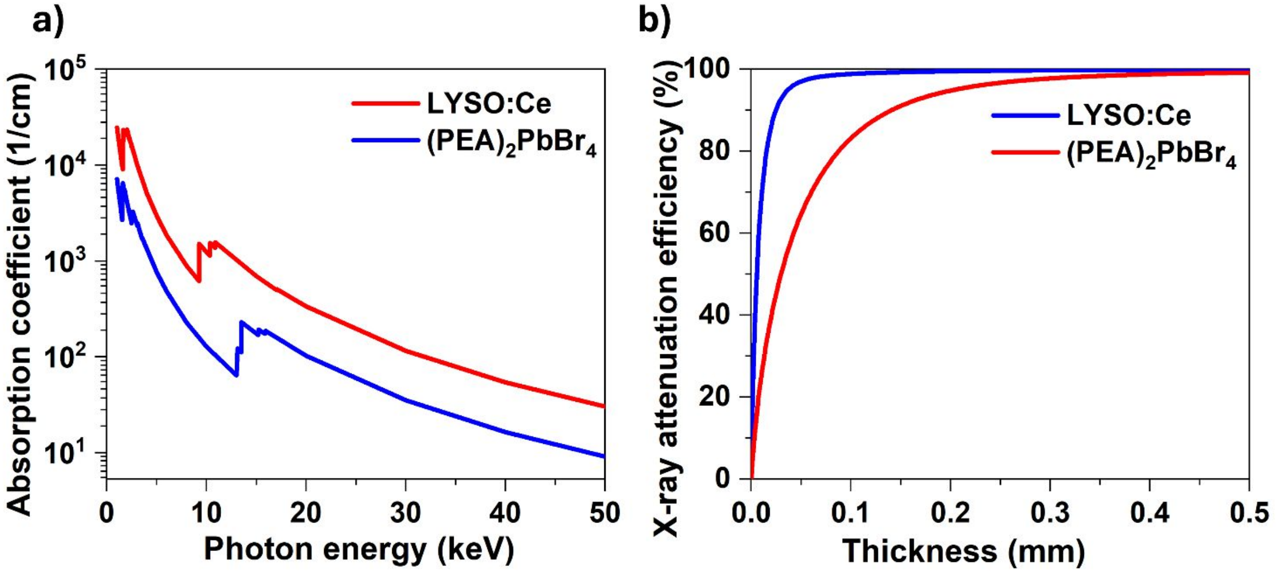

Figure S5: Absorption coefficient and attenuation efficiency of $(PEA)_2PbBr_4$ and LYSO:Ce. **a)** Absorption coefficients as a function of photon energy. **b)** X-ray attenuation efficiency as a function of film thickness for a 50 kV X-ray source.

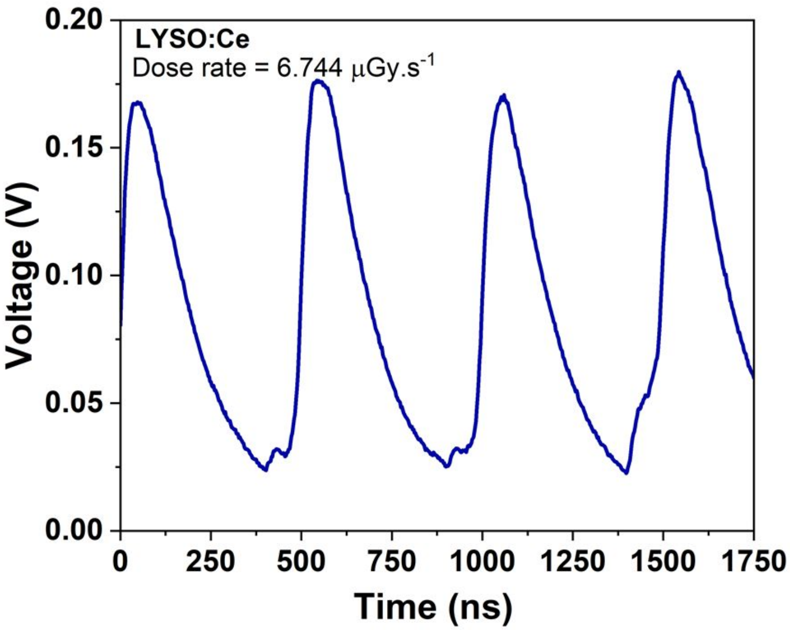

Figure S6: Detection limit of the LYSO:Ce/PMMA scintillator film. At minimum X-ray tube settings (40 kV, 0.1 μA), corresponding to a dose rate of 6.744 μGy s^{-1}, the scintillator-coupled SiPM produces a peak signal of approximately 150 mV, demonstrating reliable detection at low-dose conditions.

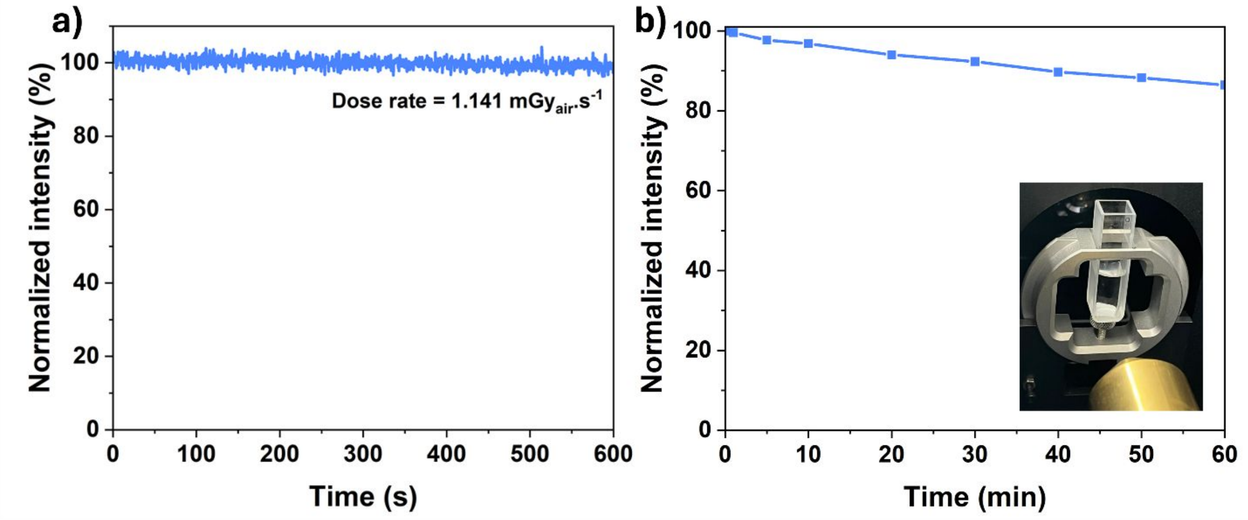

Figure S7: Stability evaluation of the $(PEA)_2PbBr_4$/PMMA composite films. (a) Radioluminescence intensity retention of the composite film as a function of accumulated delivered dose, showing approximately 98.9% of the initial intensity preserved after exposure exceeding 600 mGy, indicating high radiation stability. **(b)** Humidity stability test of the composite film under continuous irradiation (40 kV, 80 μA) while submerged in water. After 1 h of exposure, the film retains approximately 86% of its initial radioluminescence intensity, demonstrating strong environmental resilience.

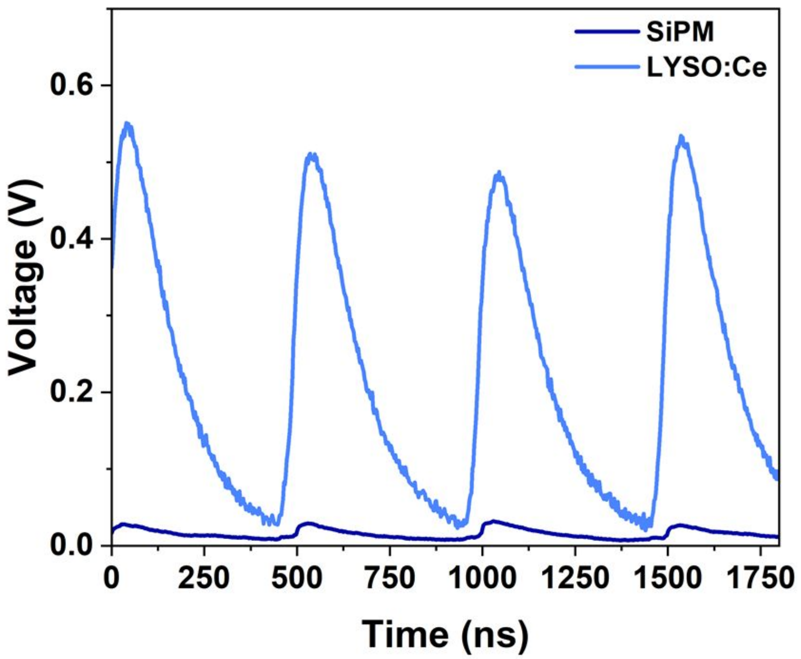

Figure S8: Response of SiPM with and without scintillator. Comparison under identical pulsed X-ray conditions (40 kV, 1.5 μA, 500 ns spacing) shows that coupling the SiPM to the LYSO:Ce/PMMA film increases the signal amplitude by 20-fold as compared to the SiPM response without scintillator.

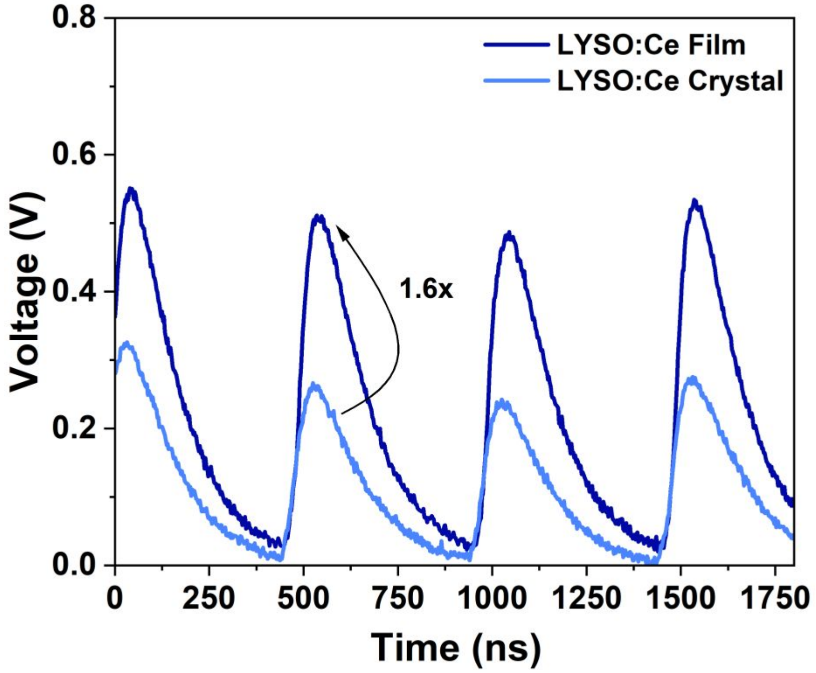

Figure S9: Performance comparison LYSO:Ce/PMMA composite film vs bulk LYSO:Ce crystal. The composite film exhibits approximately 1.6× higher peak voltage than the crystal, demonstrating enhanced photon extraction and improved light collection efficiency in the inhomogeneous structure.

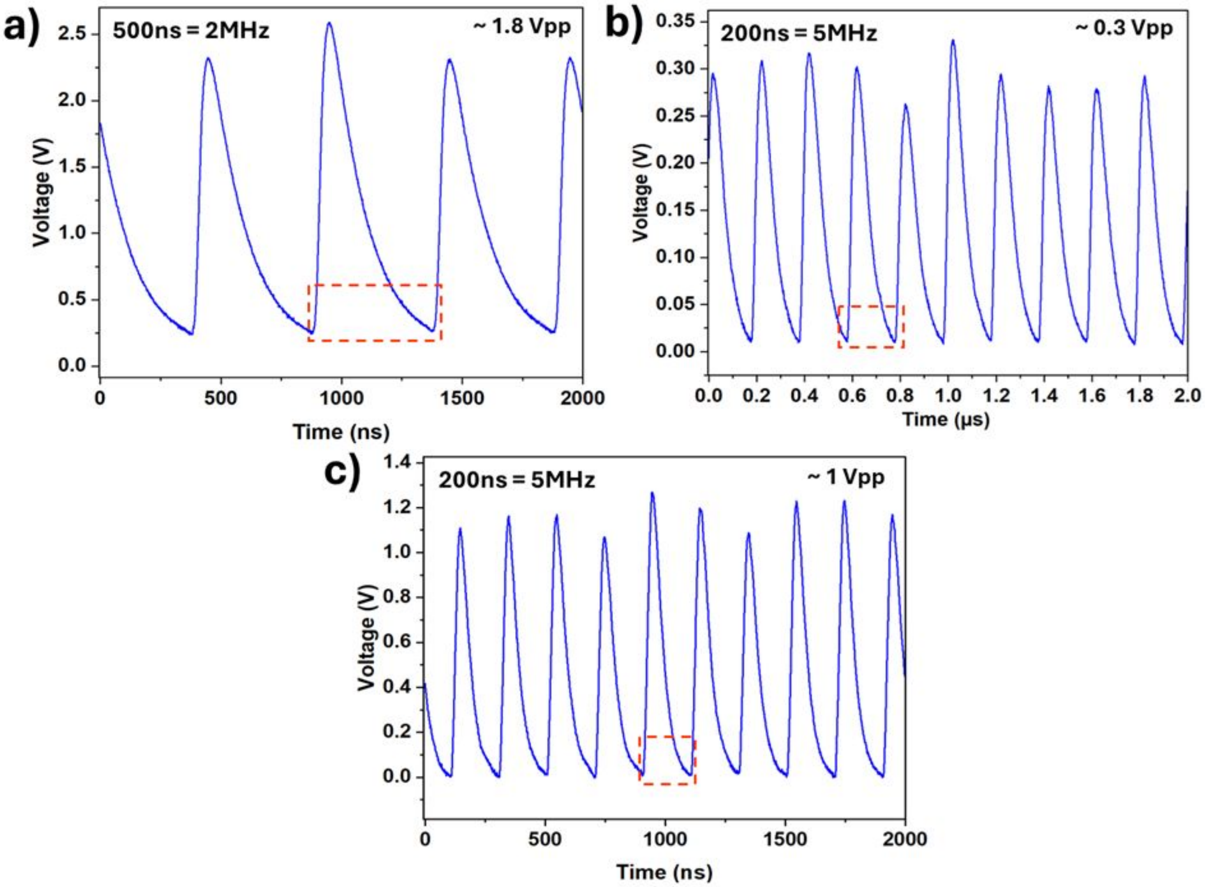

Figure S10: Selection of SiPM. a) The recharge time of the SiPM is 95ns compared to the 6ns decay time of the $(PEA)_2PbBr_4$, /PMMA, and increased deadtime of the electronics. **b)** LYSO/PMMA doesn't fully decay due to a lower recharge time of SiPM is 23ns, and risks of pile-up. **c)** At 5 MHz, the output voltage signal for $(PEA)_2PbBr_4$ is ~1Vpp.

Table S1: Quantitative Comparison of High-Frequency Radiation Detection Technologies

Ref.	Technology	Upper Detection Frequency	Dead Time / Timing	Detector Volume / Form Factor	Light Output / Dynamic Range	Cost Category
**1**	**Geiger–Müller (GM) Pulse Counter**	~10^3–10^4 counts/s	~100–300 μs intrinsic dead time (tube dependent)	Cylindrical gas-filled tube (cm-scale)	No proportional light output (binary pulse mode; no energy resolution)	Low-Moderate
**2**	**AGIPD (European XFEL)**	4.5 MHz burst-mode frame rate	220 ns pulse spacing	Large-area hybrid pixel detector (~10 × 10 cm modules; 200 μm pixel pitch)	Single-photon sensitivity up to >10^4 photons/pixel dynamic range	Very high (facility-scale instrument)
**3**	**JUNGFRAU Detector**	2 kHz nominal frame rate	Frame-based acquisition	Hybrid pixel detector (75 μm pitch; cm-scale modules)	Linear count rate capability ~20 MHz/pixel	Very high (large scientific facility instrument)
4	**JUNGFRAU Detector**	~150 kHz burst mode (16 memory cells)	Burst acquisition architecture	Hybrid pixel detector (75 μm pitch; cm-scale modules)	Linear count rate capability ~20 MHz/pixel	Very high (large scientific facility instrument)
**5**	**Bulk LYSO:Ce + Analog Readout**	System-dependent; limited by pulse pile-up	~40 ns scintillation decay constant	mm-cm rigid crystal	~25,000-33,000 photons/MeV	Moderate–High (crystal cost significant)w
*	**This Work**	Up to 5 MHz demonstrated (200 ns spacing)	~20 ns effective system dead time	Thin composite films (sub-mm thickness)	Composite-dependent; optimized for fast digital counting	Low-Moderate (scalable materials + compact electronics)

REFERENCES

1. Knoll, G. F., *Radiation detection and measurement*. John Wiley & Sons: 2010.

2. Allahgholi, A.; Becker, J.; Bianco, L.; Delfs, A.; Dinapoli, R.; Goettlicher, P.; Graafsma, H.; Greiffenberg, D.; Hirsemann, H.; Jack, S., AGIPD, a high dynamic range fast detector for the European XFEL. *Journal of Instrumentation* **2015,** *10* (01), C01023-C01023.

3. Smith, J. H.; Mozzanica, A.; Schmitt, B., Technical Design Report. **2015**.

4. Sikorski, M.; Ramilli, M.; de Wijn, R.; Hinger, V.; Mozzanica, A.; Schmitt, B.; Han, H.; Bean, R.; Bielecki, J.; Bortel, G., First operation of the JUNGFRAU detector in 16-memory cell mode at European XFEL. *Frontiers in Physics* **2023,** *11*, 1303247.

5. Li, C.; Li, Y.; Xi, J.; Xiang, S.; Hu, K., Coincidence time resolution of radiation detector based on 6×6 mm2 ToF SiPM detectors with different readout schemes. *Frontiers in Physics* **2023,** *Volume 11 - 2023*.
